# Differential fecal microbiota are retained in broiler chicken lines divergently selected for fatness traits

**DOI:** 10.1038/srep37376

**Published:** 2016-11-23

**Authors:** Qiangchuan Hou, Lai-Yu Kwok, Yi Zheng, Lifeng Wang, Zhuang Guo, Jiachao Zhang, Weiqiang Huang, Yuxiang Wang, Li Leng, Hui Li, Heping Zhang

**Affiliations:** 1 Key Laboratory of Dairy Biotechnology and Engineering Building, Inner Mongolia Agricultural University, Hohhot, 010018, China; 2 Key Laboratory of Chicken Genetics and Breeding, Ministry of Agriculture, Northeast Agricultural University, Harbin 150030, P.R. China

## Abstract

Our study combined 16S rRNA-pyrosequencing and whole genome sequencing to analyze the fecal metagenomes of the divergently selected lean (LL) and fat (FL) line chickens. Significant structural differences existed in both the phylogenic and functional metagenomes between the two chicken lines. At phylum level, the FL group had significantly less *Bacteroidetes*. At genus level, fourteen genera of different relative abundance were identified, with some known short-chain fatty acid producers (including *Subdoligranulum, Butyricicoccus, Eubacterium, Bacteroides, Blautia*) and a potentially pathogenic genus (*Enterococcus*). Redundancy analysis identified 190 key responsive operational taxonomic units (OTUs) that accounted for the structural differences between the phylogenic metagenome of the two groups. Four Cluster of Orthologous Group (COG) categories (Amino acid transport and metabolism, E; Nucleotide transport and metabolism, F; Coenzyme transport and metabolism, H; and Lipid transport and metabolism, I) were overrepresented in LL samples. Fifteen differential metabolic pathways (Biosynthesis of amino acids, Pyruvate metabolism, Nitrotoluene degradation, Lipopolysaccharide biosynthesis, Peptidoglycan biosynthesis, Pantothenate and CoA biosynthesis, Glycosaminoglycan degradation, Thiamine metabolism, Phosphotransferase system, Two-component system, Bacterial secretion system, Flagellar assembly, Bacterial chemotaxis, Ribosome, Sulfur relay system) were identified. Our data highlighted interesting variations between the gut metagenomes of these two chicken lines.

Host genetic background is a major determinant factor that controls the phenotype. The gut microbiota is recognized as an important environmental factor that interacts with its host through metabolic exchange and contributes to host energy absorption^1^. The present work investigated the host gut metagenome of a divergently selected chicken model based on fatness traits. Strong reasons have supported us to perform this work in broiler chicken.

Firstly, poultry are important protein sources in human diet and hence they are of enormous economic value; and domesticated chickens are the most common domestic animals in the world. The formation of close symbiotic relationship between the host-gut microbiota is known to be crucial in sustaining host health. Accumulating evidence shows that gut dysbiosis is linked to a variety of diseases, especially metabolic-associated syndromes like obesity^2^, diabetes^3^, and rheumatoid arthritis^4^. One vital function of the gut microbiota is to provide microbial-based metabolic pathways that are otherwise not indigenously encoded within the host genome; and in turn the gut microbiota participates in regulating the host nutritional metabolism, including nutrient assimilation and energy capture. Moreover, they act in protecting the host from pathogens, detoxification, and immune development^5^. Taking into account the crucial health-related roles, without any doubt the gut microbiota can be regarded as a potential target for improving the general health, growth performance and productivity of broiler chickens, which have always been of intense interest to breeders.

Secondly, numerous studies have concluded that the gut microbiota is linked with host obesity and energy metabolism^1^,^6^,^7^, potentially via the connection between the gut microbes and body fat absorption and disposition^8^,^9^. For instance, the phylum, *Firmicutes*, is more abundant in obese than lean individuals, and vice versa for *Bacteroidetes*. Moreover, the relative abundance of *Bacteroidetes* increases, accompanied with a decrease in *Firmicutes*, after a weight loss program for obese individuals^1^. By transferring the gut microbiota from obese or lean mice to germ-free mice, it was shown that a high *Firmicutes* to *Bacteroidetes* gut microbial ratio increased body fat accumulation^10^. Apart from bacteria, the dominant human gut archaeon, *Methanobrevibacter smithii*, affects host calorie harvest and adiposity through the digestion of dietary polysaccharides^11^. In contrast, the reduction of some normal gut bacteria along with the enrichment of certain opportunistic pathogens in the gut microbiota may raise the risk of weight gain. The opportunistic pathogen, *Enterobacter cloacae* B29, isolated from obese human’s gut is able to induce obesity and insulin resistance in germ-free mice^12^.

However, most available data in this aspect are based on rodents and human models^10^,^13^,^14^, which may not be completely suited in the case of chicken because of its unique anatomy and physiological functioning. Chickens swallow food and store it in the crop before digestion and absorption taking part in the gizzard, small intestine and cecum. Only a relatively short time of 2.5 hours is required for the food to pass through the upper intestine, contrasting to that of 12–20 hours in the ceca^5^. The prolonged retention of digesta in chicken ceca, where the largest quantity and diversity of microbes are hosted along the chicken gastrointestinal tract^15^, may indicate the essential nutritional function of the locality, including nutrient absorption, digestion of non-start polysaccharides (often present in high proportion in feed), recycling of nitrogen via uric acid dissimilation, and provision of B vitamins and essential amino acids^5^,^16^.

Additionally, most currently published studies only described the structure and function of the chicken gut microbiota, and the spatial and temporal changes upon specific stimulations^15^,^17^,^18^,^19^,^20^. Our study compared and contrasted the gut metagenome chickens selectively bred based on fatness traits. Selective breeding is a common agricultural method that allows preferential selection of desirable genetic traits. This work has taken the advantage of the availability of two broiler lines^21^, the fat (FL) and lean (LL) chicken, which have been selectively bred for 15 generations (at the time of this experiment) from the parental Arbor Acres broilers based on the plasma very low density lipoprotein (VLDL) concentration and abdominal fat percentage (AFP). The LL chickens are characterized by a high efficiency in extracting and incorporating food source energy into lean meat, while the FL chickens have a great tendency of abdominal fat deposition. Polymorphisms of several gene loci may be responsible for the fatness traits in the divergent chicken lines, including the insulin-like growth factor binding protein 2^22^, adipocyte fatty acid-binding protein^23^, acetyl-CoA carboxylase α^24^, and the PC1/PCSK1 region of the Z chromosome^25^. The divergent chicken lines also displayed variations in the preadipocyte microRNA expression profile^26^ and specific genes (namely SLC9A3, GNAL, SPOCK3, ANXA10, HELIOS, MYLK, CCDC14, SPAG9, SOX5, VSNL1, SMC6, GEN1, MSGN1 and ZPAX) within the copy number variation regions^27^.

We hypothesized that, apart from the host genetics, variation of the fatness trait may also link with the composition of the gut microbial metagenome. Thus, in this study, we compared the faecal microbial metagenome of these two chicken lines by integrating the 16S rRNA-pyrosequencing with whole genome sequencing (WGS). We aimed to investigate whether the process of lean and fat chicken selection simultaneous led to colonization of different spectra of gut microbiota. Data generated from this study have provided interesting insights into the role of environmental factors, e.g. the gut microbiota, in relation to the host phenotype in a genetically-predisposed model.

## Materials and Methods

### Ethics statement

All animal work was conducted according to the guidelines for the care and use of experimental animals established by the Ministry of Science and Technology of the People’s Republic of China (Approval number: 2006–398) and approved by the Laboratory Animal Management Committee of Northeast Agricultural University and the Ethical Committee of the Inner Mongolia Agricultural University.

### Animals and sample collection

Two chicken lines, FL and LL, were provided by the Northeast Agricultural University. These two chicken lines were both originated from Arbor Acres broiler, which underwent 15 generations of selection since 1996 based on the VLDL and AFP at 7 weeks of age. Briefly, the VLDL and AFP for all the first generation male chickens were measured at 7 weeks of age. The VLDL and AFP of the next generation were measured and compared to that of the previous one, and broilers of lower or higher average VLDL and AFP were chosen for subsequent breeding, as described previously^28^. In this way, the chicken lines were divergently selected for 15 generations. At 15^th^ generations, the AFP of the FL was on average 4.57 higher than that of the LL (Supplementary Fig. S1 and Supplementary Table S1). Each chicken was raised in an individual cage in order to prevent contamination from uncontrolled particle intake and feathers^29^. All birds had free access to water. Birds and environmental controls were checked twice daily by trained staff. The temperature was maintained at 16–18 °C, and the humidity was maintained at 50–60%. At different life stages, the birds were fed with different diets, according to the Arbor Acres Plus parent stock breeder management guide and nutrition specifications (http://en.aviagen.com/). At the time of sampling (bird age between week 37 to 40), the birds were feed-restricted on a standard diet containing 14.2% crude protein and metabolic energy of 2745 kcal/kg. The feed nutritive content and digestible amino acid supplementation at the time of sampling are provided in Table 1.

Fecal samples of each chicken (15 lean and 14 fat line chickens) were collected by laying clean papers on the cage floor, and the droppings were then transferred to 5 ml tube by 1000 μL pipette. All of the 29 samples were used for bacterial 16S rRNA genes V1-V3 region pyrosequencing and for whole-genome shotgun (WGS) sequencing.

### DNA extraction

Genomic DNA was extracted from each fecal sample using Qiagen DNA Stool Mini Kit (Qiagen, Hilden, Germany) following the instructions of the manufacturer. The quality of extracted DNA was assessed by 0.8% agarose gel electrophoresis and spectrophotometry (optical density at 260 nm/280 nm). All extracted DNA samples were stored at −20 °C for further analysis.

### PCR Amplification and 16S rRNA pyrosequencing

The V1-V3 region of the 16S ribosomal RNA (rRNA) genes in all the samples was amplified by PCR for barcoded pyrosequencing. For PCR amplification, 10 ng of extracted DNA was amplified in a 20 μL reaction buffer containing 4 μL 5× FastPfu Buffer, 2 μL 2.5 mM dNTPs, 0.4 μL FastPfu polymerase, 0.8 μL of each 5 μM primer (TransGen Biotech) and double deionized water. The primers used to amplify the bacterial V1-V3 region of the 16S rRNA gene were 27 F (5′-AGAGTTTGATCCTGGCTCAG-3′) and 533 R (5′-TTACCGCGGCTGCTGGCAC-3′). The PCR program was as follows: 94 °C for 5 min; 30 cycles of 94 °C for 45 s, 55 °C for 40 s, and 72 °C for 1 min; and a final extension of 72 °C for 7 min). The primers used for amplifying archaeal 16S rRNA gene were Arch344F (5′-ACGGGGYGCAGCAGGCGCGA-3′) and Arch915 (5′-GTGCTCCCCCGCCAATTCCT-3′). The PCR program was as follows: 95 °C for 2 min; 30 cycles of 95 °C for 30 s, 55 °C for 30 s, and 72 °C for 30 s with a final extension of 72 °C for 5 min.

The quality of the PCR products was ensured using Agilent 2100 Bioanalyzer (Agilent Technologies, Palo Alto, Calif.) in accordance with the manufacturer’s instructions. The PCR products were pooled in equimolar ratios with a final concentration of 100 nmol/L for pyrosequencing (Roche GS FLX), performed by Shanghai Majorbio Bio-pharm Technology Co., Ltd.

### Bioinformatics processing of 16S rRNA pyrosequencing data

The extraction of high-quality sequences was performed with the QIIME package (Quantitative Insights Into Microbial Ecology) (v1.7). Raw sequences were selected based on sequence length, quality, primer and tag, according to the following criteria: the length of the raw sequences was more than 300 bp; the variable region was more than 300 bp in length and lying between the two primers; the read sequence had a perfect match with the barcode; the high-quality score (>20) for the proportion of bases was larger than 93% in the raw read.

PyNAST and UCLUST were applied to align sequences under 100% clustering of sequence identity in order to obtain unique representative sequences. Operational taxonomic units (OTUs) were classified under the threshold of 97% identity by using UCLUST. FastTree was applied to construct the *de novo* phylogenetic tree. Ribosomal Database Project (RDP) Classifier (Release 11.4) was applied to assign taxonomic to each OTU representative sequence. Owing to the relatively short length of the V1-V3 region of 16S rRNA genes, BLASTN from the NCBI website was applied when RDP failed to classify an OTU taxonomically. Differently defined thresholds were set to assign the reference sequence into the matching taxonomic group, which were 95%, 92%, 91%, 85%, and 75% for genus, family, order, class, and phylum, respectively^30^.

### Whole-genome shotgun (WGS) sequencing and quality control

All samples were sequenced with the Illumina HiSeq2000 instrument. Libraries were prepared with a fragment length of approximately 300 bp. Paired-end reads were generated with 100 bp in the forward and reverse directions. The length of each read was trimmed with Sickle. Reads that aligned to the chicken genome were also removed. This set of high-quality reads was then used for further analysis. The sample taxonomic profile was also determined directly from the metagenome dataset using two online software, i.e. Parallel-META^31^ and MetaPhlAn^32^, following the instructions of the two software developers.

### Illumina short reads *de novo* assembly, gene prediction and construction of the non-redundant gene catalogue

The microbial Illumina reads were assembled into contigs using IDBA-UD^33^ with default parameters. Genes were predicted on the contigs with MetaGeneMark^34^. A non-redundant gene catalogue was constructed with CD-HIT^35^ using a sequence identity cut-off of 0.95, with a minimum coverage cut-off of 0.9 for the shorter sequences.

### Functional annotation

Putative amino acid sequences, translated from the gene catalogue, were aligned against the proteins/domains in the Cluster of Orthologous Group (COG) and Kyoto Encyclopedia of Genes and Genomes (KEGG) databases (submit online) using BLASTP (e-value ≤ 1e-5 with a bit score higher than 60). Each protein was assigned to the KEGG orthologue group (KO) or COG by the highest scoring annotated hit.

### Computation of relative gene abundance

To assess the relative gene abundance, reads were aligned against the gene catalogue with Bowtie2^36^ using parameters: -p 12 -x nt -1 R1.fastq -2 R2.fastq -S R.sam. Then, to normalize the sequencing coverage, the relative abundance instead of the raw read count was used to quantify the gut microbial genes. The calculation process was as follows:

Step 1: Calculation of the copy number of each gene:


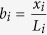


Step 2: Calculation of the relative abundance of gene i:


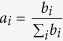


*a*_*i*_: the relative abundance of gene i.*b*_*i*_: the copy number of gene i from sample N.*L*_*i*_: the length of gene i.*x*_*i*_: the number of mapped reads.

The relative gene abundances and other calculation were completed using Python scripts.

### Statistical analyses

Statistical analyses were performed mainly using R packages (http://www.r-project.org/), together with Python^37^, Canoco for Windows 4.5 (Microcomputer Power, NY, USA), and PAST^38^. Rarefaction analysis, Shannon diversity index and Simpson’s diversity index were used to estimate the richness and diversity of OTUs. Principal coordinate and principal component analyses (PCoA and PCA) were used to assess the gut microbiota and metagenome structure of different samples, respectively. Redundancy analysis (RDA) was applied to identify bacterial groups which significantly contributed to the structural difference. The relative abundances of differential taxonomic groups were visualized by heatmap in R using the “pheatmap” package. Differences in the relative abundances of taxonomic groups and at gene level between samples were evaluated with Mann-Whitney test. False discovery rate (FDR) values were estimated using the Benjamini–Yekutieli method to control for multiple testing^39^. P-values less than 0.05 were considered statistically significant. Differential genes were also tested with DESeq2^40^. Significantly differentiating KEGG modules were identified according to the final reporter score calculated from the aggregated Z-score of individual KOs with the cut-off level of ≥1.6 (90% confidence according to normal distribution)^41^. Whether the significantly differentiating modules were enriched in the FL or LL chickens was further determined by comparing the number of individual KOs that was enriched in the specific chicken line.

### Nucleotide sequence accession numbers

Datasets generated by the shotgun and 16S rRNA amplicon sequencing approaches reported in this study have been deposited in the GenBank database (accession number: SRP083441) and MG-RAST project (ID: 19742), respectively.

## Results

### Sequence abundance and diversity of 16S rRNA gene

A total of 263,158 of v1-v3 region of 16S rRNA raw sequence reads were generated from 29 chickens (15 LL and 14 FL), with an average of 9074 sequence read for each sample. 46,415 sequence reads were delimited through PyNAST alignment and 100% sequence identity clustering for further analysis. 7312 OTUs were identified at 97% sequence similarity level with high threshold identity and with an average of 788 OTUs for each sample. The lowest level of taxon abundance for each OTU was determined by combining homologous sequence alignment method and clustering which based on information extracted from RDP and Greengenes (Release 13.5) databases. Results showed that 19.6% of sequences were unable to be assigned to the genus level. The Shannon diversity curves for all samples plateaued (Supplementary Fig. S2), even though the individual rarefaction curves did not reach saturation phase. This suggested that the increasing of sequencing depth would possibly identify more new phylotypes, but the current analysis had already captured the majority of the microbial diversity, respectively.

The Shannon and Simpson indices were applied to evaluate the diversity of gut microbiota, while the chao1 and observed species indices were indicators for species abundance. No significant difference was found between lean and fat chickens for all four indices, as assessed by Mann-Whitney test (Supplementary Table S2), and p-values for Shannon, Simpson, chao1 and observed species were 0.15, 0.18, 0.39 and 0.47.

### Core gut microbiota detected in all samples based on 16S rRNA amplicon sequencing

In this study, the ‘core gut microbiota’ in chicken was defined as the taxonomical units (genus and OTUs) that were detected in all samples. Nine core genera, including *Clostridium* (23.44%), *Bacteroides* (18.78%), *Lactobacillus* (8.77%), *Ruminococcus* (3.97%), *Hallella* (1.61%), *Subdoligranulum* (1.07%), *Faecalibacterium* (1.04%), *Roseburia* (0.98%), and *Eubacterium* (0.31%), were identified (Supplementary Table S3).

### Comparison of gut bacterial composition between LL and FL at phylum and genus levels based on 16S rRNA amplicon sequencing

Nineteen phyla were identified within the complete dataset. The 4 most dominant phyla (*Firmicutes, Bacteroidetes, Proteobacteria* and *Actinobacteria*) accounted for 99.11% of the total sequence, contributing to 53.44%, 41.09%, 3.22% and 1.33% for LL and 71.36%, 23.40%, 3.43% and 0.98% for FL, respectively. The proportion of *Bacteroidetes*, but not the other 3 dominant phyla, were significantly different between the LL and FL samples (LL>FL, 41.09% versus 23.40%, p = 0.034). Minor phyla, including *Thermotogae, Verrucomicrobia, Cyanobacteria, Acidobacteria, Fibrobacteres, Chloroflexi, Gemmatimonadetes, Synergistetes, Fusobacteria, TM7, Deinococcus-Thermus, Tenericutes, Deferribacteres, Lentisphaerae, Spirochaetes*, together contributed to 0.1% of the total sequence.

**Figure 1:**
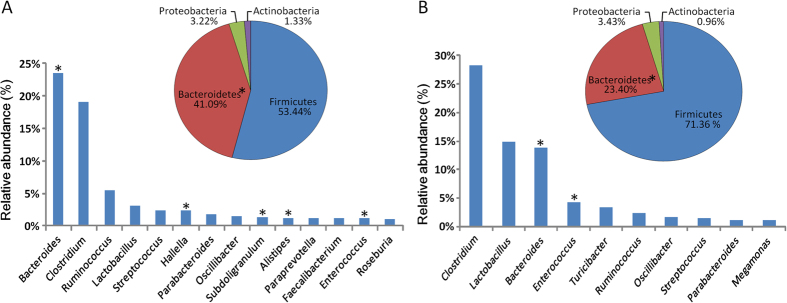
The dominant and subdominant phyla and genera (i.e. >1% relative abundance) in lean (**A**) and fat (**B**) line chickens. ‘*’ Represents the significant differing phyla and genera between the two groups (P-values of less than 0.05).

The dominant gut microbiota genera mainly belonged to the phyla, *Firmicutes* and *Bacteroidetes* (Fig. 1). However, considerable difference was noted between the fecal microbiota of the two chicken lines. In the gut microbiota of lean chickens, the relatively abundant genera (>1%) of *Firmicutes* included *Clostridium* (19.06%)*, Ruminococcus* (5.50%), *Lactobacillus* (3.09%), *Streptococcus* (2.39%), *Oscillibacter* (1.52%), *Subdoligranulum* (1.30%), *Faecalibacterium* (1.18%)*, Enterococcus* (1.14%), *Roseburia* (1.03%), whereas the *Bacteroidetes* was comprised with a wider diversity of relatively abundant genera, namely *Bacteroides* (23.45%), *Hallella* (2.36%), *Parabacteroides* (1.79%), *Alistipes* (1.23%), *Paraprevotella* (1.21%) (Fig. 1A). For FL chickens, the dominant and subdominant genera of over 1% relative abundance included members of the *Firmicutes (Clostridium, Lactobacillus, Enterococcus, Turicibacter, Ruminococcus, Oscillibacter, Streptococcus, Megamonas) of* 28.14%, 14.86%, 4.26%, 3.30%, 2.32%, 1.68%, 1.40%, and 1.02%, respectively) and *Bacteroidetes (Bacteroides* and *Parabacteroides* of 13.77% and 1.13%, respectively) (Fig. 1B).

Moreover, the proportions of 5 out of the 9 identified core genera were significantly higher in LL as compared to FL, including *Ruminococcus, Subdoligranulum, Eubacterium, Bacteroides, Hallella* (p values ranged from 0.001 to 0.047). Other genera which were also significantly enriched in LL included *Alistipes, Butyricicoccus, Hespellia, Anaerosporobacter, Odoribacter, Prevotella, Collinsella* and *Blautia* (p values ranged from 0.003 to 0.05). In contrast, the proportions of *Enterococcus* and *Corynebacterium* were lower in LL (p = 0.022, 0.045, respectively) (Supplementary Table S4).

### Multivariate analysis of the gut microbiota structure of LL and FL chickens based on 16S rRNA amplicon sequencing

**Figure 2:**
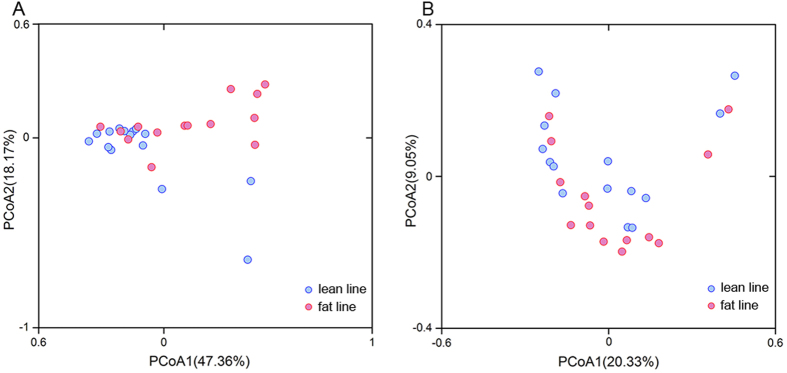
Principal coordinate analysis based on (**A**) weighted and (**B**) unweighted UniFrac metric distance.

In order to compare the overall difference of the gut microbiota of LL and FL chickens, PCoA was performed. The PCoA score plots based on weighted and unweighted UniFrac are shown in Fig. 2. Although sample overlapping occurred between the two samples groups, clustering tendencies were observed, suggesting a moderate difference existed in the gut microbiota structure between the LL and FL chickens. Results from the multivariate analysis of variance (MANOVA) revealed the significant difference of gut microbiota between LL and FL (p < 0.05). Moreover, clustering analysis based on the weighted (p = 0.0024), but not unweighted (p = 0.7767), UniFrac distances revealed significant difference between the two chicken lines (Supplementary Fig. S3), indicating that the difference lied on the more prevalent lineages rather than the rare taxonomical groups.

**Figure 3:**
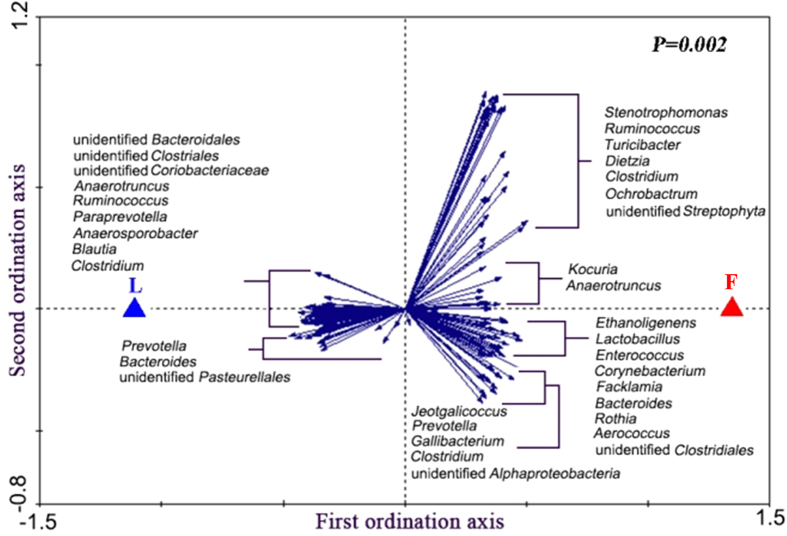
Biplot of redundancy analysis (RDA) on the basis of OTU relative abundance at 98% similarity level. ‘
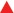
’ and ‘
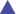
’ represent the constrained explanatory variables, fat (F) and lean (L) lines. Blue arrows represent response variables with the first ordination axis explaining for at least 9.8% of the variability. A p-value of 0.002 was generated from Monte Carlo Permutation Test.

**Figure 4:**
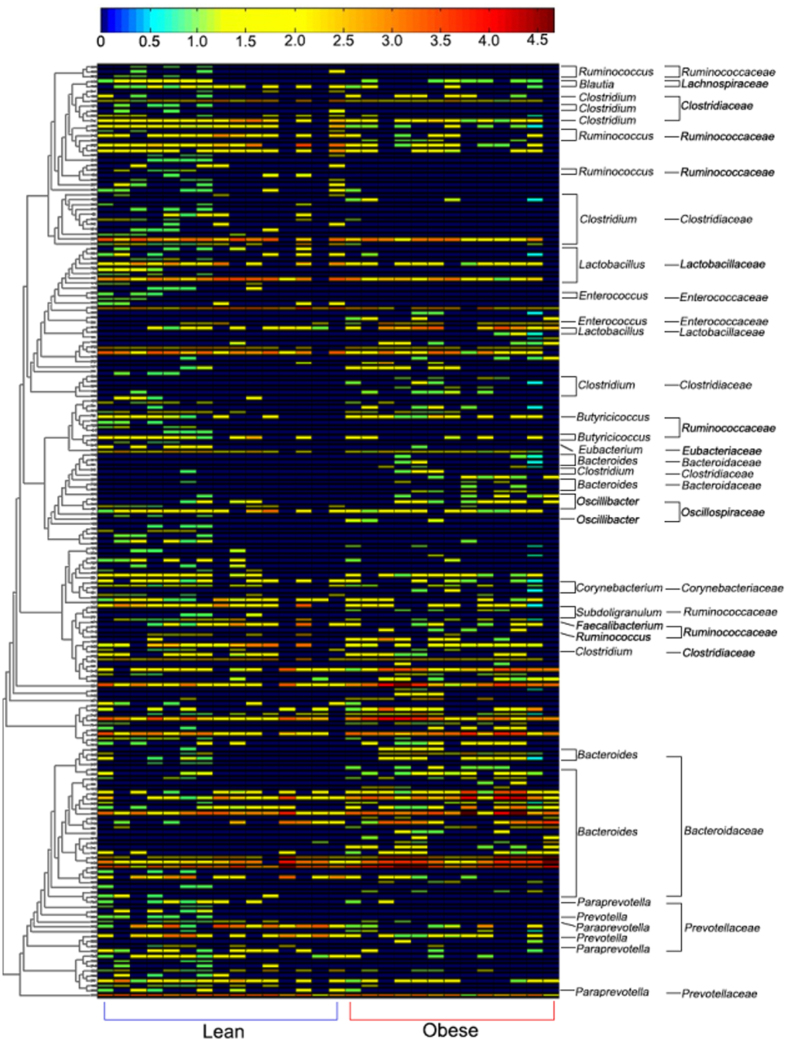
Heatmap showing the relative abundances of the 190 key variables differentiating the microbiota of lean and obese chickens. Significantly different bacterial groups are shown at the right side of the heatmap. Phylogenetic tree built based on the 190 OTUs is at the left side.

To further identify the specific bacterial groups principally accounting for the difference observed between the LL and FL gut microbiota, RDA was carried out. In this case, the LL and FL chickens were the “nominal environmental variables”, and the relative abundances of all OTUs (at 97% similarity) were the response variables. Monte Carlo Permutation Test showed that the constrained ordination model by lean or obese was statistically significant (p = 0.002) and the first canonical axis was able to explain 9.8% of the variability of response variables. Moreover, a total of 190 OTUs were identified as key variables which had good correlation with sample scores on the RDA canonical axis. Among them, 109 and 81 OTUs were enriched in the gut microbiota of LL and FL chickens, respectively (Fig. 3). The heatmap constructed by the relative abundance of these 190 OTUs is shown in Fig. 4.

As assessed by Mann-Whitney test, 49 (18 *Bacteroidaceae*, 6 *Ruminococcaceae*, 6 *Prevotellaceae*, 5 *Lachnospiraceae*, 3 *Clostridiaceae*, 11 from other or unidentified families) out of the 190 key OTUs were significantly more abundant in lean chickens (p = 0.048–0.002), whereas the relative abundance of 47 OTUs (14 *Clostridiaceae*, 5 *Lactobacillaceae*, 4 *Ruminococcaceae*, 3 *Micrococcaceae*, 3 *Erysipelotrichaceae*, 18 from other or unidentified families) were significantly enriched in obese chickens (p = 0.046–0.002) (Supplementary Table S5).

### Comparison of the gut archaeal composition between LL and FL

Archaeal members were also identified in the gut microbiota of both LL and FL chickens (Table 2). All archaeal sequences could be assigned to the phylum *Euryarchaeota.* At the genus level, most sequences belonged to *Methanobrevibacter* and *Methanocorpusculum*, and a few of the sequences correspond to *Methanosarcina*. Each of the 3 genera occupied less than 0.1% of the total sequences. No significant difference was found in the archeal composition between the two sample groups.

To investigate the archaea in the gut of both LL and FL, two steps UCLUST method combining with multivariate statistics were applied to conduct OTUs. By PCoA, the first two principal coordinates for weighted UniFrac were 86.09% and 5.78%, and 35.57% and 37.30% for unweighted UniFrac. Furthemore, the two groups overlapped each other; thus, no obvious difference in the gut archaea profile existed between the two groups (p > 0.05) (Supplementary Fig. S4).

### Coverage of WGS sequencing

To profile the gut microbial metagenome of the chickens, WGS sequencing was performed on all 29 samples. However, one of them failed the quality test (data not shown); thus, no data was included in the metagenome analysis from this sample. The 28 samples yielded a total of 234.4 Gb of pair-end reads (an average of 52,485,882 high-quality reads and 239,690 genes per sample) that were of high-quality and were free of chicken DNA and adaptor contaminants (Supplementary Table S6).

### Structures and functions of the chicken gut microbial metagenome

**Figure 5:**
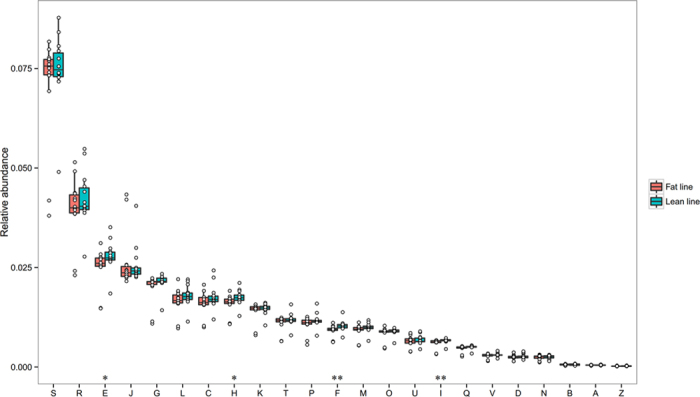
Comparison of the structure of lean and fat chicken gut metagenomes. (A) Differential abundance of COG functional categories of the two chicken lines. COG category codes are as follows: A, RNA processing and modification; B, Chromatin structure and dynamics; C, Energy production and conversion; D, Cell cycle control, cell division, chromosome partitioning; E, Amino acid transport and metabolism; F, Nucleotide transport and metabolism; G, Carbohydrate transport and metabolism; H, Coenzyme transport and metabolism; I, Lipid transport and metabolism; J, Translation, ribosomal structure and biogenesis; K, Transcription; L, Replication, recombination and repair; M, Cell wall/membrane/envelope biogenesis; N, Cell motility; O, Posttranslational modification, protein turnover, chaperones; P, Inorganic ion transport and metabolism; Q, Secondary metabolites biosynthesis, transport and catabolism; R, General function prediction only; S, Function unknown; T, Signal transduction mechanisms; U, Intracellular trafficking, secretion, and vesicular transport; V, Defense mechanisms; W, Extracellular structures; Y, Nuclear structure; Z, Cytoskeleton. P-values of <0.01 and <0.05 are represented by ‘**’ and ‘*’, respectively.

**Figure 6:**
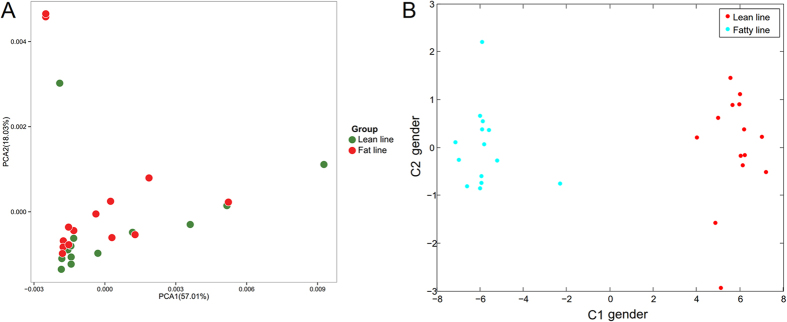
Principal component analysis (**A**) and multivariate analysis of variance (**B**) of the gut metagenomes of lean and fat line chickens based on KEGG orthology distribution.

The structure and function of the LL and FL gut microbial metagenome were analyzed based on the COG and KEGG functions. The relative abundances of all the COG functional groups in the LL and FL chickens are represented by box plots (Fig. 5). Four of the functional categories were significantly different between the two chicken lines (all with relative abundance of lean > fat line), which were Amino acid transport and metabolism (E) (p = 0.0350), Nucleotide transport and metabolism (F) (p = 0.0042), Coenzyme transport and metabolism (H) (p = 0.0186), and Lipid transport and metabolism (I) (p = 0.0079), respectively. To visualize the functional difference between the two sample groups, PCA analysis was performed based on all detected KO (Fig. 6A) (with PC1 and PC2 representing 57.01% and 18.03% of the total variance). Although only a weak clustering pattern was observed on the score plot with mild overlap of symbols representing the two groups, further analysis by MANOVA revealed that they were significantly different (p = 0.0012) (Fig. 6B).

### Differential chicken gut microbial KEGG genes, modules and pathways

A total of 6739 KEGG genes could be assigned from the whole dataset, among which 320 genes (179 and 141, representing 2.66% and 2.09%, overrepresented in lean and fat line chickens with p-values ranging from 0.0003 to 0.0491 and 0.0010 to 0.0499, respectively) were differentially enriched, as detected by pairwise Mann-Whitney test (Supplementary Table S7).

To understand the biological meaning of such gene abundance differences, all the detected genes were further assigned to KEGG module and pathway levels. The detected genes could be assigned to 579 different KEGG modules. Forty (6.91%) of the modules had a final reporter score of over 1.6 (cutoff limit for significantly differentiating pathway). By comparing the number of individual KOs that was enriched in the specific chicken line, 31 (5.35%) and 8 (1.38%) modules were found to be more abundant in the lean and fat line chickens, respectively, while 1 (0.12%) module displayed similar level of activity between the two chicken lines (Supplementary Table S8). Table 3 includes only the modules with a high (≥ 80%) or a low (≤ 20%) proportion of KO that had higher relative gene abundance in lean line.

Similarly, based on the same level of final reporter score cutoff, a total of 15 differential pathways could be identified; most of them were relating to the category of Metabolism (namely Biosynthesis of amino acids, Pyruvate metabolism, Nitrotoluene degradation, Lipopolysaccharide biosynthesis, Peptidoglycan biosynthesis, Pantothenate and CoA biosynthesis, Glycosaminoglycan degradation, Thiamine metabolism), followed by Environmental Information Processing (namely Phosphotransferase system (PTS), Two-component system, Bacterial secretion system), Cellular Processes associated with Cell motility (Flagellar assembly, Bacterial chemotaxis), and Genetic Information Processing (Ribosome, Sulfur relay system) (Supplementary Table S9). To verify the profiles of differential pathways and modules, a second method, DESeq2, was used to compare the functional metagenomes of the two sample groups. Comparing to the results generated by Mann-Whitney test, highly similar results were obtained at both KEGG pathway (Supplementary Table S10) and module levels (Supplementary Table S11).

## Discussion

Our study analyzed the gut microbiome of the divergently selected lean and fat broiler chicken lines. We asked whether different spectra of gut microbiota will be retained in these two chicken lines; and if so, whether the two spectra of gut microbiota potentially carry genomes that confer different metabolic capacity.

To answer our first question, we comparatively analyzed the fecal microbiota profiles of the two chicken lines mainly based on the 16S rRNA amplicon sequencing. Our MANOVA analysis revealed a significant difference between the LL and FL chicken fecal microbiota structure. At phylum level, significantly more *Bacteroidetes* (p = 0.0343) was found in the LL samples. *Bacteroidetes* is known to be associated with fat accumulation in chickens^42^ and less of these bacteria are present in obese human individuals^1^,^43^.

At genus level, some of the 14 identified differential genera are known short-chain fatty acid (SCFA)-producers. The butyrate-producers (*Subdoligranulum, Butyricicoccus, Eubacterium*)^44^,^45^, propionate-producers (*Bacteroides*)^46^ and acetate-producers (*Blautia*)^47^,^48^ were diminished in the FL samples. *Subdoligranulum* and *Butyricicoccus* can stimulate the growth of intestinal epithelial cells and thus reduce the invasion and colonization of *Salmonella* in veterinary medicine^49^. *Eubacterium* is a possible butyrate-producer in animal guts^44^. Low grade inflammation is involved actively in obesity development; and butyrate is anti-inflammatory and protects the intestinal barrier function^50^,^51^. Thus, fewer gut butyrate-producers in the FL group may contribute to its fat accumulation.

Meanwhile, *Enterococcus* sequences were approximately 4-fold more abundant in the FL samples. *Enterococcus* is reported to be associated with colorectal cancer^52^,^53^; and *Enterococcus faecalis* can damage eukaryotic cellular DNA in colonic epithelial cells by producing extracellular superoxides and hydroperoxides^54^,^55^. The local cellular damages and reactive oxygen species caused by these bacteria may alter intestinal permeability, lead to cell death, and hence act as triggers for inflammation and obesity.

No significant difference existed in the relative abundance of *Lactobacillus* between LL and FL samples, but an apparent variation existed (3.09% ± 0.92% versus 14.86% ± 6.82% in LL and FL samples, respectively). Moreover, ten fatness traits correlated key responsive OTUs identified by RDA belonged to *Lactobacillus*. In all cases, more were present in the FL samples, with OTUs 7838, 9029, and 8615 exhibiting significant differences (p-values = 0.003–0.014; 16.64-, 12.65-, and 4.31-fold enrichment in FL samples). Probiotics have been used in agricultural practices to increase growth rate, improve digestion, nutrient absorption and nutrient digestion. The probiotics-driven weight gain effect is strain-specific. The administration of *Lactobacillus fermentum, Lactobacillus ingluviei, Lactobacillus agilis* and *Lactobacillus salvarius* was associated with weight gain in chickens^56^,^57^,^58^,^59^, while consuming some other *Lactobacillus* species resulted in weight loss in rodents or humans^60^,^61^,^62^. The mechanism of *Lactobacillus* in host weight modification is complex and yet to be elucidated.

Generally, more *Methanobrevibacter* was found in the FL group, though the difference was not statistically significant. Methanogenic archaea, in particular *Methanobrevibacter*, participate in regulating gut metabolism by removing excessive bowel hydrogen. This subsequently improves the efficiency of microbial fermentation and enhances host energy capture. In a gnotobiotic mouse model, *Methanobrevibacter* results in weight gain in the presence of *Bacteroides thetaiotaomicron*^12^. *Methanobrevibacter* improve acetate and butyrate production meanwhile eliminate hydrogen and formate^63^, which are vital carbon sources for colon epithelium cells. This syntrophism between the gut bacteria and archaea may raise energy extraction when indigestible polysaccharide diets are given. The higher *Methanobrevibacter* abundance in the FL group may contribute to its obese phenotype.

Apart from describing the sample microbiota using a more traditional approach based on 16S rRNA amplicon sequencing, we also extracted the taxonomic profiles directly from the metagenome dataset by using two publicly accessible online tools, Parallel-META and MetaPhlAn (Supplementary Figs S5 and S6). We found some discrepancies between the phylogenic metagenome profiles as determined by the three methods; such discrepancies are possibly caused by the differences in the algorithm used in taxonomic assignment in each case^64^, as well as bias between the metagenomic- and 16S rRNA-based sequencing approaches^65^,^66^,^67^. The current study detected a broader range of bacterial genera with the 16S rRNA amplicon sequencing approach (246 versus 88 and 90 different bacterial genera with Parallel-META and MetaPhlAn, respectively) (data not shown), although many of these genera comprised only a minute proportion of the entire bacterial microbiota community.

To answer our second question, we analyzed the functional fecal metagenomes of the two chicken lines. Even though symbols representing the two groups mildly overlapped in the PCA plot based on KEGG orthology distribution (Fig. 6A), further MANOVA analysis confirmed that structural difference existed between the two KO-annotated functional metagenomes. Sequences encoding for the Amino acid transport and metabolism (E), Nucleotide transport and metabolism (F), Coenzyme transport and metabolism (H), and Lipid transport and metabolism (I) were overrepresented in the COG annotation of LL dataset.

Annotation of KEGG KO revealed a total of 15 differential pathways. Many of these KEGG pathways potentially relate to obesity, adiposity and energy balance regulation. For example, Lipopolysaccharide biosynthesis and Flagellar assembly are indirectly involved in host adiposity. Obesity is considered to be connected with chronic low-grade inflammation. Obesity causes noticeable structural alterations at the gut lining, resulting in elevated intestinal permeability that favors the gut microbiota-derived lipopolysaccharide translocation to the bloodstream. The endotoxemia may activate toll-like receptor 4 to proinflammatory status^68^. Flagellin is the major flagellar structural protein, which is specifically detected by the host toll-like receptor 5. Mice deficient of toll-like receptor become hyperphagic and develop obesity; and the transfer of gut microbiota from these mice to wild type germ-free mice confer metabolic syndrome features to the recipients, suggesting that the Flagellar assembly plays a role in obesity development^69^. On the other hand, peptidoglycan exerts an anti-inflammatory effect via the peptidoglycan recognition protein 3^70^, which may counter the low-grade inflammation in obesity status. Some other pathways associate with nutrient absorption (including Biosynthesis of amino acids, Pyruvate metabolism and Phosphotransferase system (PTS)) and bacterial colonization and proliferation (including Two-component system, Bacterial chemotaxis, Bacterial secretion system). These pathways are required for substrate sensing and foraging, as well as carbohydrate substrate transporting and utilizing; thus, they are crucial for energy extraction and provision.

Only few reports have discussed the roles of vitamin metabolism in obesity development. The administration of thiamine prevents from obesity and metabolic disorders in Otsuka Long-Evans Tokushima Fatty rats that resemble human metabolic syndrome and obesity^71^, while pantothenic acid derivatives were found to exert hypolipidemic effect to hypothalamic obesity mice induced by aurothioglucose, potentially by the mechanisms of insulin resistance reduction and lipolysis in serum and adipose tissue^72^. The functions of other differential pathways in obesity development are less clear (e.g. Ribosome, Sulfur relay system, Nitrotoluene degradation, Glycosaminoglycan degradation).

Microbiome-derived SCFAs are considered as key players in regulating host obesity. Generally, butyrate and propionate are antiobesogenic, while acetate is obesogenic^73^. At KEGG module level, of particular interest is the higher abundance of lysine and isoleucine biosynthesis pathways of the LL group. Some gut commensal bacteria (including members of *Eubacterium*) are able to produce butyrate from lysine, although no gut microbe is known to contain the complete pathway^74^. Several amino acids, including both lysine and isoleucine, can serve as precursors for gut butyrogenesis^75^. Thus, we speculate that the gut microbiota of LL chicken may produce butyrate using amino acids as substrates.

At KEGG module level, two methanogenesis (M00357 and M00567) and the Pyridoxal biosynthesis (M00124) modules were enriched in the FL group, which may contribute to fat accumulation. The higher activity of methanogenesis is in line with the higher relative abundances of methanogenic archaea in the FL samples. A recent study found that the active form of vitamin B6 (pyridoxal 5-phosphate) is linked with adipogenesis using a comparative metabolomic approach^76^.

On the other hand, the ascorbate biosynthesis module (M00129) is enriched in the LL sample. Ascorbic acid supplementation may suppress adiposity and insulin resistance gene expression in high-fat diet induced obese rats^77^. Such effect may due to the intrinsic ascorbate anti-oxidative activity that ameliorates free radical-induced oxidative stress and thus lessens inflammation.

To sum up, we highlighted the overall structural differences between the fecal phylogenic and functional metagenomes of the LL and FL chickens. Between the two groups, significant differences were found in the relative abundances of some energy metabolite-related bacteria (especially the SCFA-producers) and potential pathogens (e.g. *Enterococcus*), and in numerous biochemical pathways relating to obesity, adiposity and energy balance. Our experimental design does not allow us to draw a concrete conclusion on whether the microbiota residing in the FL or LL chicken gut significantly or at least partially contributed to obesity or, alternatively, the modified metabolism driven by the host fatness traits selection resulted in modulated intestinal tract environment and thus the shift of microbiota composition to adapt to host obesity. Nevertheless, this study has provided a deeper insight into the possible contribution of gut microbiota in modulating obesity.

## Additional Information

**How to cite this article**: Hou, Q. *et al*. Differential fecal microbiota are retained in broiler chicken lines divergently selected for fatness traits. *Sci. Rep.* **6**, 37376; doi: 10.1038/srep37376 (2016).

**Publisher's note:** Springer Nature remains neutral with regard to jurisdictional claims in published maps and institutional affiliations.

## Supplementary Material

Supplementary Figures

Supplementary Tables

## Figures and Tables

**Table 1:** The nutritive value of chicken feed.

Nutritive value	
Crude protein (%)	14.2
Metabolic energy (ME) (kcal/kg)	2745
**Digestable amino acids (%)**	
Lysine	0.61
Methionine & cystine	0.45
Methionine	0.57
Threonine	0.27
Valine	0.51
Isoleucine	0.41
Arginine	0.13
Tryptophan	0.48

**Table 2:** The relative abundance of archaea of the two chicken lines.

Genus	p-value	Average (%)		Median, range (%)	
		LL	FL	LL	FL
*Methanobrevibacter*	0.255	25.19	47.82	6.82, 0.12–73.43	56.58, 0.04–97.41
*Methanocorpusculum*	0.310	71.88	48.64	89.46, 24.57–97.96	37.91, 2.30–99.72

**Table 3:** Significant differential KEGG modules in lean or fat chicken lines.

Modules	Final reporter score	Proportion of KO with gene abundance of lean>fat line	Identity of the differential module
Modules that were enriched in lean line chicken			
M00207	1.64	80%	Environmental information processing | Saccharide, polyol, and lipid transport system | Putative multiple sugar transport system
M00002	1.67	91%	Carbohydrate and lipid metabolism | Central carbohydrate metabolism | Glycolysis, core module involving three-carbon compounds
M00050	1.72	83%	Nucleotide and amino acid metabolism | Purine metabolism | Guanine ribonucleotide biosynthesis IMP = >GDP, GTP
M00323	1.73	100%	Environmental information processing | Phosphate and amino acid transport system | Urea transport system
M00491	1.74	80%	Environmental information processing | Saccharide, polyol, and lipid transport system | arabinogalactan oligomer/maltooligosaccharide transport system
M00237	1.76	100%	Environmental information processing | Phosphate and amino acid transport system | Branched-chain amino acid transport system
M00061	1.81	86%	Carbohydrate and lipid metabolism | Other carbohydrate metabolism | D-Glucuronate degradation
M00260	1.86	80%	Genetic information processing | DNA polymerase | DNA polymerase III complex, bacteria
M00016	1.94	83%	Nucleotide and amino acid metabolism | Lysine metabolism | Lysine biosynthesis, succinyl-DAP pathway, aspartate => lysine
M00096	2.10	89%	Carbohydrate and lipid metabolism | Terpenoid backbone biosynthesis | C5 isoprenoid biosynthesis, non-mevalonate pathway
M00525	2.10	100%	Nucleotide and amino acid metabolism | Lysine metabolism | Lysine biosynthesis, acetyl-DAP pathway, aspartate = >lysine
M00360	3.39	90%	Metabolism | Aminoacyl tRNA | Aminoacyl-tRNA biosynthesis, prokaryotes
M00359	3.54	100%	Metabolism | Aminoacyl tRNA | Aminoacyl-tRNA biosynthesis, eukaryotes
M00178	5.22	96%	Genetic information processing | Ribosome | Ribosome, bacteria
Modules that were enriched in fat line chicken			
M00210	1.63	20%	Environmental information processing | Saccharide, polyol, and lipid transport system | Phospholipid transport system
M00194	1.76	20%	Environmental information processing | Saccharide, polyol, and lipid transport system | Maltose/maltodextrin transport system
M00331	1.80	20%	Environmental information processing | Bacterial secretion system | Type II general secretion system
M00357	2.15	16%	Energy metabolism | Methane metabolism | Methanogenesis, acetate = >methane
M00567	2.49	4%	Energy metabolism | Methane metabolism | Methanogenesis, CO2 = >methane

Remarks: The table only includes modules that have a high (≥ 80%) or a low (≤ 20%) proportion of KO of gene abundance of lean>fat line, indicating that these modules are either enriched in the lean or fat line chickens, respectively. The complete list of significant differential modules is provided in Supplementary Table 8.
