# Supplementary material for: Differential fecal microbiota are retained in broiler chicken lines divergently selected for fatness traits
Source: Sci Rep. 2016 Nov 23;6:37376. doi: 10.1038/srep37376 (PMC5120256; doi:10.1038/srep37376)
Supplement: Supplementary Figures [file srep37376-s1.pdf]

Differential fecal microbiota are retained in broiler chicken lines divergently selected for fatness traits

Running title: Fecal microbiota in fat and lean line broiler chickens

Qiangchuan Hou[a†], Lai-Yu Kwok[a†], Yi Zheng[a], Lifeng Wang[a], Zhuang Guo[a], Jiachao Zhang[a], Weiqiang Huang[a], Yuxiang Wang[b], Li Leng[b], Hui Li[b**], Heping Zhang[a*]

[a] *Key Laboratory of Dairy Biotechnology and Engineering Building, Inner Mongolia Agricultural University. Hohhot, 010018, China*

[b] *Key Laboratory of Chicken Genetics and Breeding, Ministry of Agriculture, Northeast Agricultural University, Harbin 150030, P.R. China*

[†] These authors contributed equally to this work.

---

[*]Corresponding author: Key Laboratory of Dairy Biotechnology and Engineering, Ministry of Education, Inner Mongolia Agricultural University, Hohhot 010018, P. R. China. Tel: 0086-471-4319940. *E-mail address*: hepingdd@vip.sina.com (Heping Zhang)

[**]Co-corresponding author. Key Laboratory of Chicken Genetics and Breeding, Ministry of Agriculture, Northeast Agricultural University, Harbin 150030, P. R. China. *E-mail address*: lihui@neau.edu.cn (Hui Li)

Supplementary materials

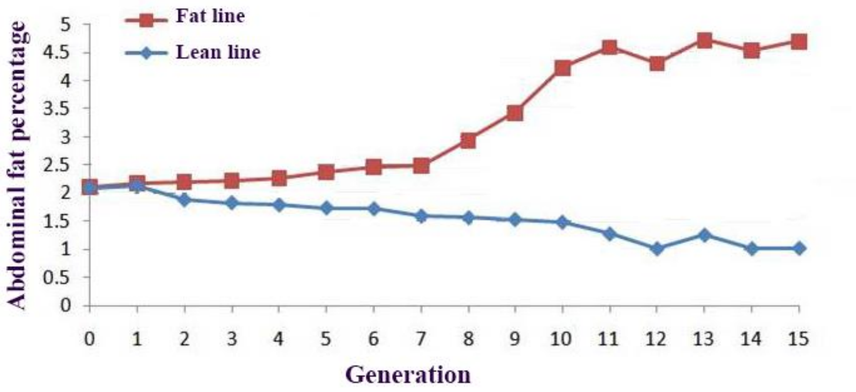

Supplementary Figure S1 The abdominal fat percentage of fat and lean line chickens during the divergent selection

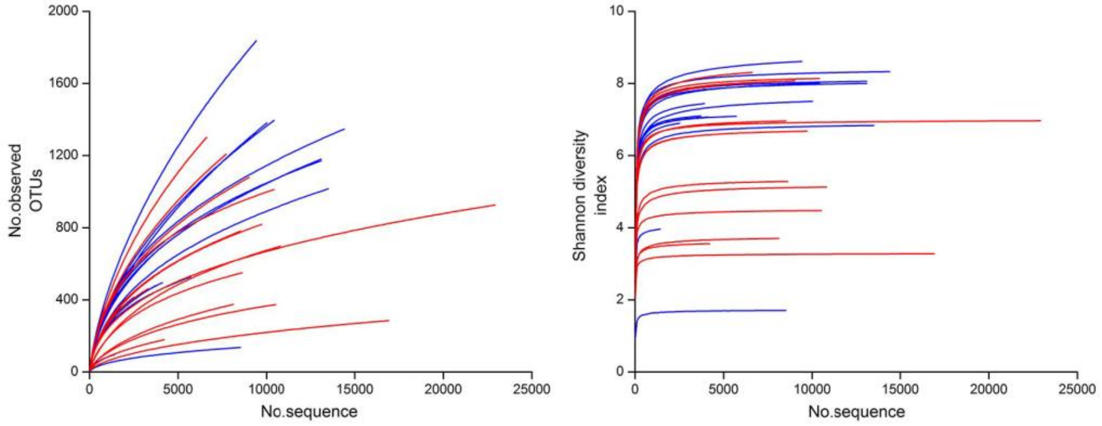

Supplementary Figure S2. Rarefaction and Shannon diversity index curves for all fecal samples

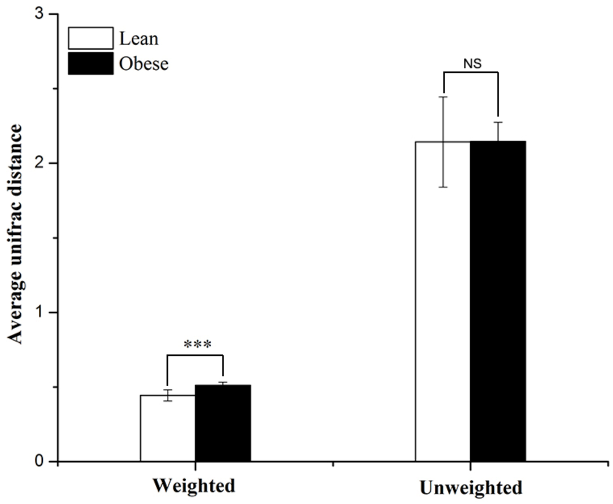

Supplementary Figure S3. Comparison between UniFrac distances of LL and FL chickens. '***' represents significant difference between sample groups at p<0.001. 'NS' means no significant difference exists.

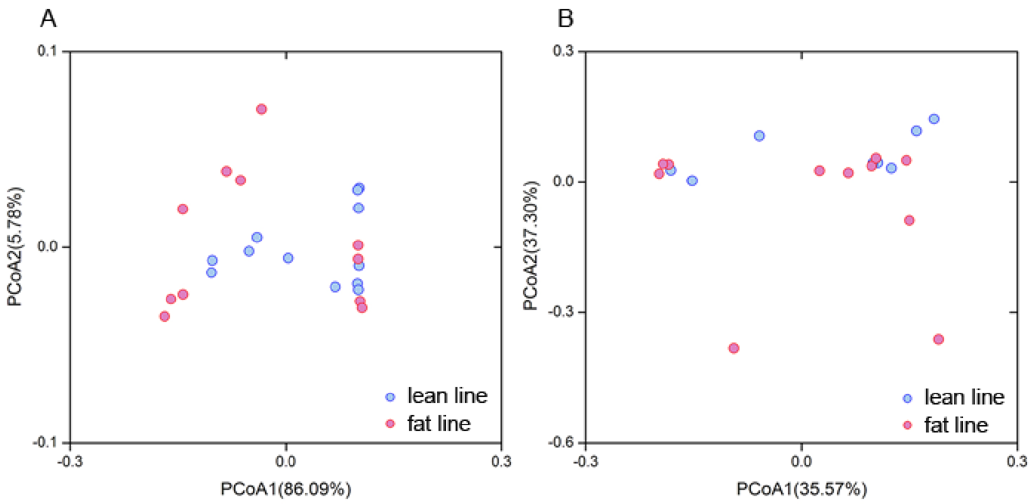

Supplementary Figure S4. Principal coordinate analysis score plot based on (A) weighted and (B) unweighted UniFrac metric distance for the relative abundance of archaea of the two groups of chickens.

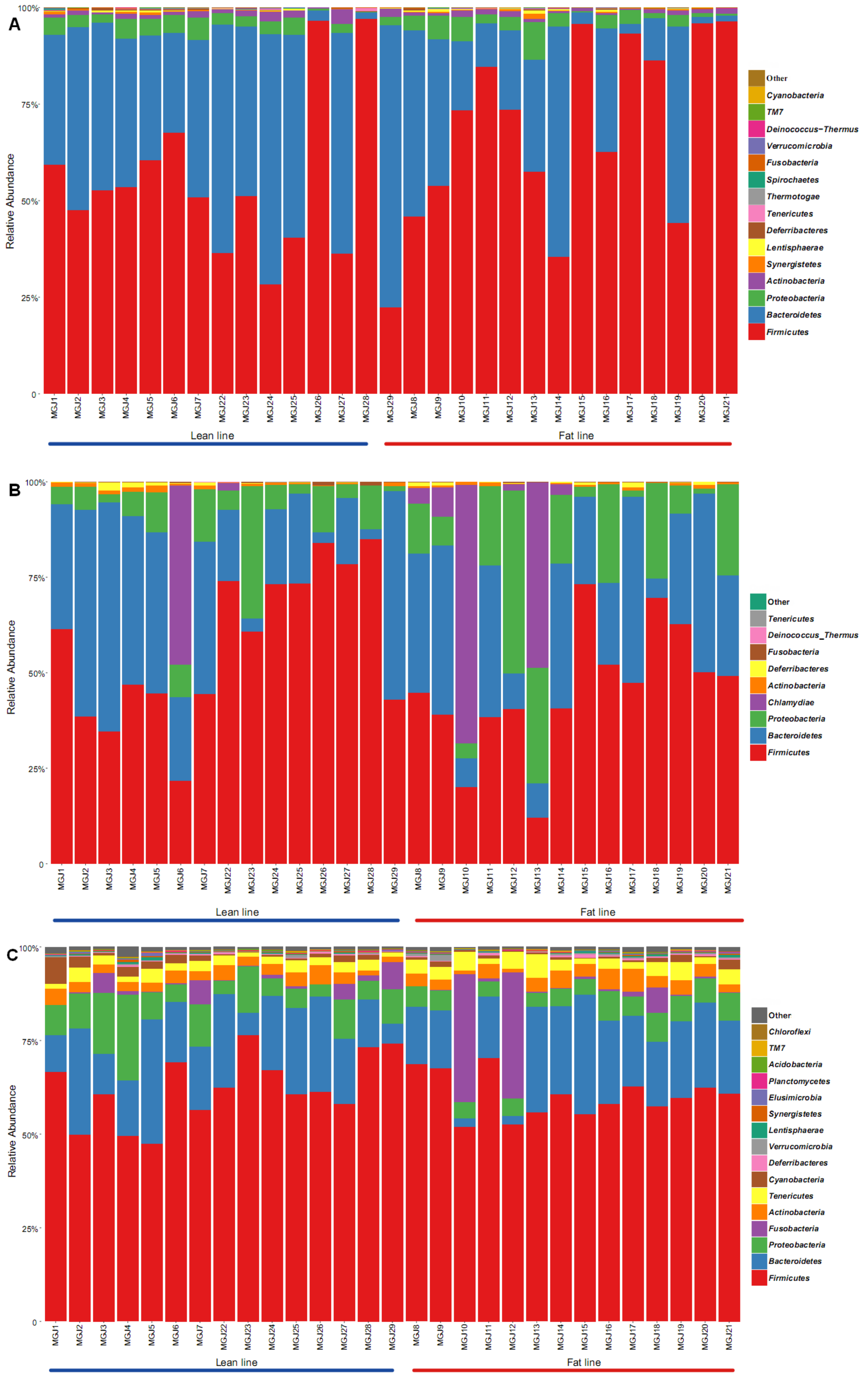

Supplementary Figure S5. Taxonomic metagenome profile at phylum level, determined by A. 16S rRNA-amplicon sequencing, B. MetaPhlAn, and C. Parallel-META.

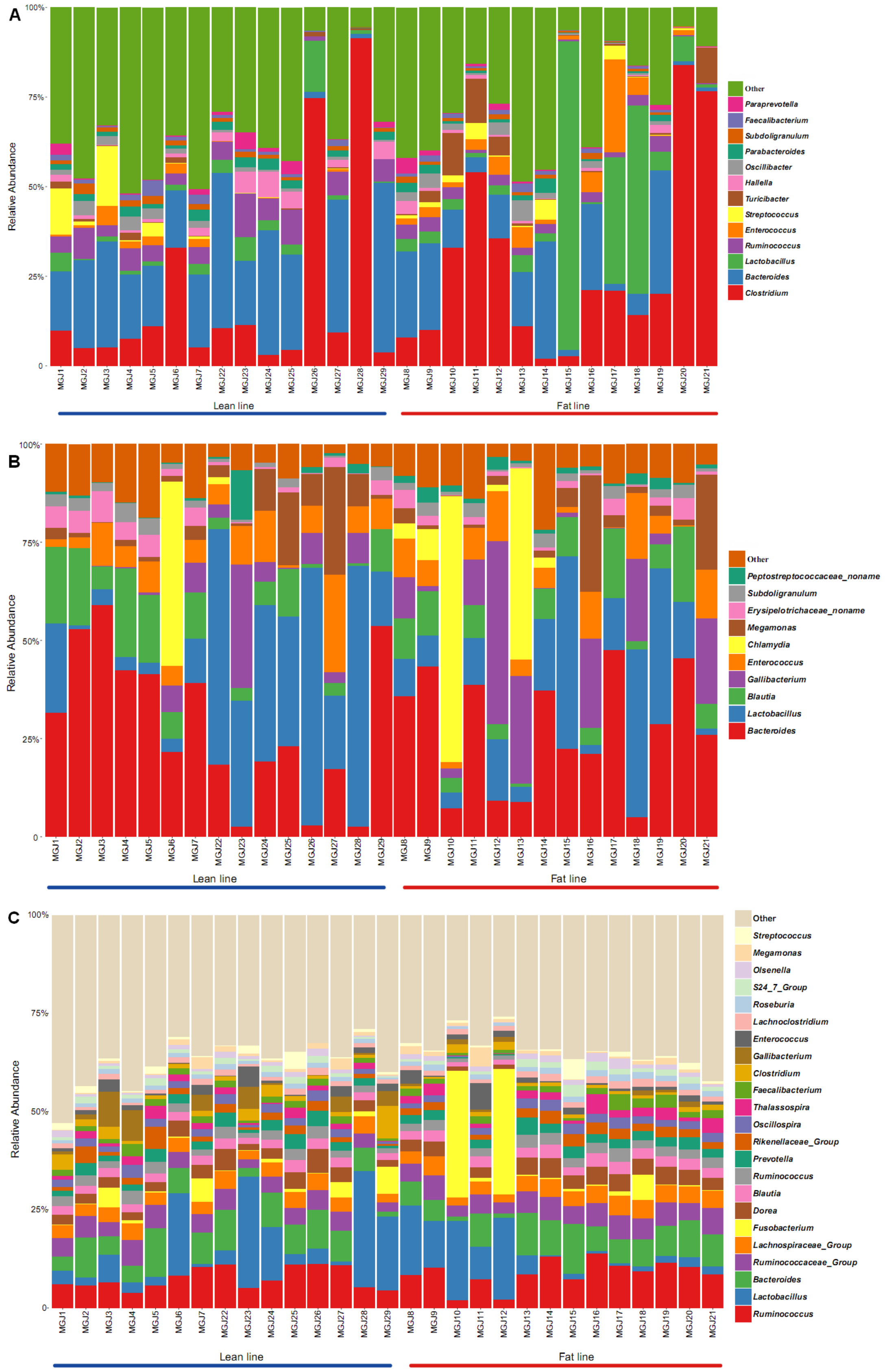

Supplementary Figure S6. Taxonomic metagenome profile at genus level, determined by A. 16S rRNA-amplicon sequencing, B. MetaPhlAn, and C. Parallel-META.

Supplementary Table S1: Body weight, abdominal fat content and very low density lipoprotein in LL and FL chickens at the 15th generation

Supplementary Table S2: Microbial diversity and abundance indices of LL and FL faecal samples based on 16S rRNA sequencing

Supplementary Table S3: Core genera that were present in all samples

Supplementary Table S4: Differential bacterial genera identified in LL and FL faecal samples based on 16S rRNA sequencing

Supplementary Table S5: Comparison of the 190 identified key OTUs between FL and LL faecal samples

Supplementary Table S6: Coverage of whole genome sequencing

Supplementary Table S7: Differential KEGG KO identified in LL and FL faecal samples based on whole genome sequencing

Supplementary Table S8: Proportion of significantly differing KEGG modules between FL and LL faecal samples

Supplementary Table S9: Differential KEGG pathways between FL and LL faecal samples

Supplementary Table S10: Differential pathways identified by Mann-Whitney Test and DESeq2

Supplementary Table S11: Differential modules identified by Mann-Whitney Test and DESeq2
